# Supplementary material for: Predicting visual function by interpreting a neuronal wiring diagram
Source: Nature. 2024 Oct 2;634(8032):113–23. doi: 10.1038/s41586-024-07953-5 (PMC11446822; doi:10.1038/s41586-024-07953-5)
Supplement: Supplementary file 6 — CRF and ERF predictions for individual TmY4 and TmY9 cells. Analogous to Supplementary Data 3, but for TmY target types. Shown are the top four monosynaptic pathways, the strongest pathway passing through each of the top ten intermediary types (ranking from Extended Data Fig. 7), and the trisynaptic pathway Tm1–TmY–Dm3–TmY (see the section entitled Prediction of spatial normalization). [file 41586_2024_7953_MOESM6_ESM.zip › DataS4/TmY9qΓèÑ/720575940606802818.pdf]

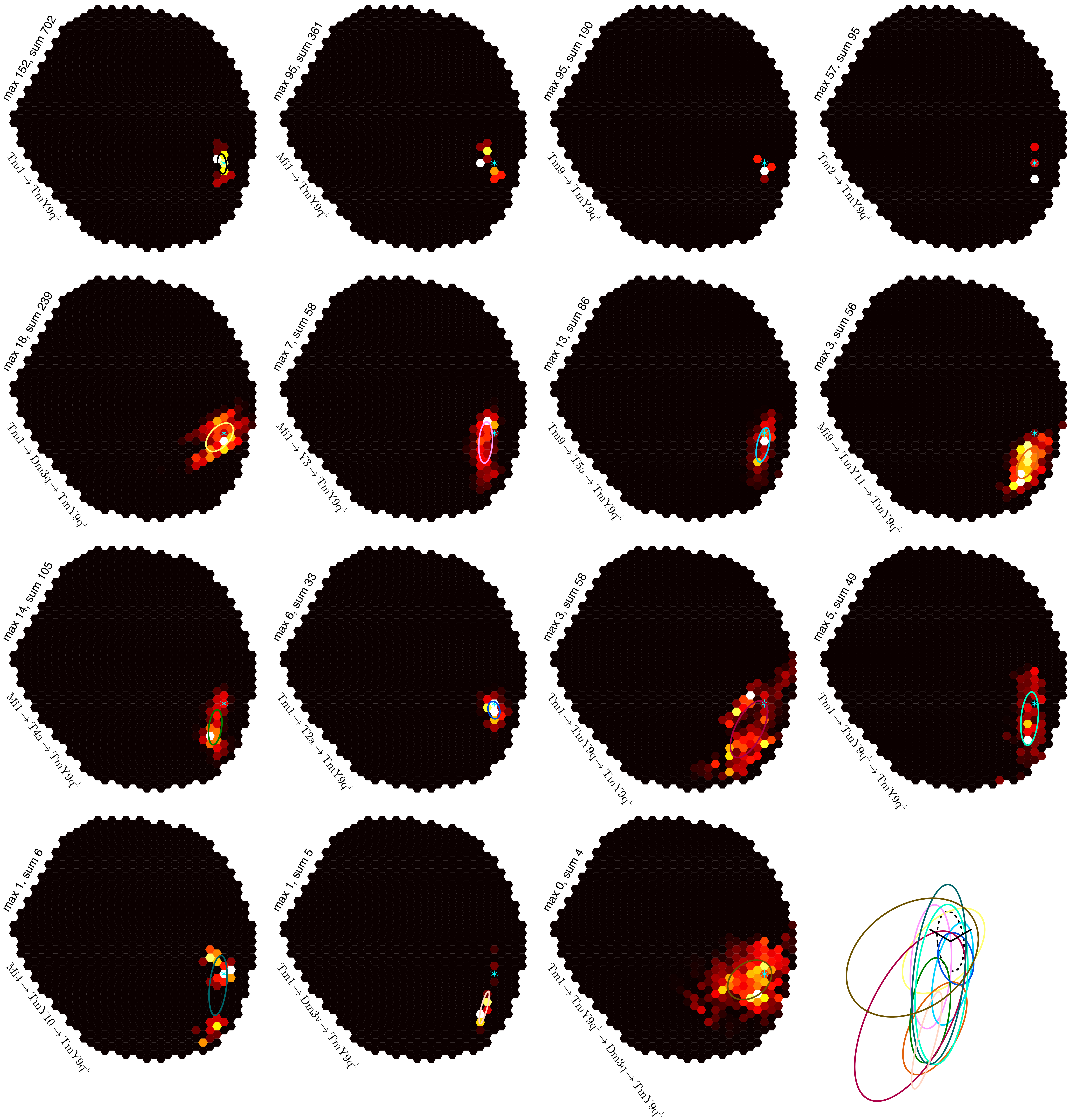
max 152, sum 702
Tm1 → TmY9q⊥
max 95, sum 361
Mi1 → TmY9q⊥
max 95, sum 190
Tm9 → TmY9q⊥
max 57, sum 95
Tm2 → TmY9q⊥
max 18, sum 239
Tm1 → Dm3q → TmY9q⊥
max 7, sum 58
Mi1 → Y3 → TmY9q⊥
max 13, sum 86
Tm9 → T5a → TmY9q⊥
max 3, sum 56
Mi9 → TmY11 → TmY9q⊥
max 14, sum 105
Mi1 → T4a → TmY9q⊥
max 6, sum 33
Tm1 → T2a → TmY9q⊥
max 3, sum 58
Tm1 → TmY9q → TmY9q⊥
max 5, sum 49
Tm1 → TmY9q⊥ → TmY9q⊥
max 1, sum 6
Mi4 → TmY10 → TmY9q⊥
max 1, sum 5
Tm1 → Dm3v → TmY9q⊥
max 0, sum 4
Tm1 → TmY9q⊥ → Dm3q → TmY9q⊥
